# Choice of speed under compromised Dynamic Message Signs

**DOI:** 10.1371/journal.pone.0243567

**Published:** 2020-12-11

**Authors:** Kaveh Bakhsh Kelarestaghi, Alireza Ermagun, Kevin Heaslip, John Rose

**Affiliations:** 1 ICF Incorporated LLC, Fairfax, Virginia, United States of America; 2 Department of Civil and Environmental Engineering, Mississippi State University, Starkville, Mississippi, United States of America; 3 The Charles Edward Via, Jr. Department of Civil and Environmental Engineering, Virginia Tech, Clemson, Virginia, United States of America; 4 Business School, University of Technology Sydney, Sydney, Australia; Tongii University, CHINA

## Abstract

This study explores speed choice behavior of travelers under realistic and fabricated Dynamic Message Signs (DMS) content. Using web-based survey information of 4,302 participants collected by Amazon Mechanical Turk in the United States, we develop a set of multivariate latent-based ordered probit models participants. Results show female, African-Americans, drivers with a disability, elderly, and drivers who trust DMS are likely to comply with the fabricated messages. Drivers who comply with traffic regulations, have a good driving record, and live in rural areas, as well as female drivers are likely to slow down under fabricated messages. We highlight that calling or texting, taking picture, and tuning the radio are distracting activities leading drivers to slow down or stop under fictitious scenarios.

## Introduction

As a communication channel between road authorities and drivers, Dynamic Message Signs (DMS) have been deployed to disseminate traffic information to drivers. The content of DMS typically publicizes information on road conditions, traffic incidents, and travel time to enable drivers to use the existing transportation system more efficiently by diverting routes, controlling speed, and reducing the occurrence of traffic conflicts [1–7]. The effectiveness of DMS in transportation is reflected in reducing the speed of the approaching traffic in work zone areas [4], harmonizing the speed of traffic at the presence of imminent speed variation [1], influencing the speed behavior of drivers under adverse weather and slippery road conditions [2,8], and diverting traffic to alternative routes [3,9]. Kolisetty et al. [8] study the speed change behavior of drivers using a driving simulator and report that DMS is effective in speed reduction under adverse weather condition information. In a similar study, Yan and Wu [10] show that drivers are likely to lower their speed as they get closer to DMS. Erke et al., [9] examine the effectiveness of DMS on speed and route choice in a field study. They assess speed measurements of 3,342 vehicles and conclude there is high compliance with DMS content.

Previous research, on the flip side, elaborates on the failure of DMS in accomplishing its tasks due to speed variation. The speed variation generally occurs because of (1) increased attention demand to react to messages and (2) the shockwave created by lead traffic [9]. Using a driving simulator, Guattari et al., [11] study the speed variation pattern of drivers at the presence of DMS. The results indicate that the speed variation is higher when drivers do not understand the content, whilst the speed profile is stable when drivers comprehend the content. Harms et al. [12] try to understand whether DMS traffic-irrelevant content disturbs the transportation network. Collecting the driving behavior of 32 participants in a driving simulator environment, they observe a significant speed reduction due to reading the DMS content. The speed variation creates impulsive driving behavior leading to closer spacing between vehicles and frequent lane-changing maneuvers, and thereby potentially causing a severe accident [13].

Cyber and physical attacks against intelligent transportation systems have been a pressing issue for transportation owners, operators, and users [14,15]. Such an attack on DMS could cause dire consequences. Although the existing literature on both effectiveness and ineffectiveness of DMS is rich, little is known about the impact of fabricated DMS content on the speed choice behavior of drivers. A recent study [16] elaborates on DMS hacking events across the United States and identify their negative impacts on the behavior of drivers. The research scrutinized cyber and physical attacks on more than two dozen DMSs to narrate the dire outcomes of a comprised DMS on traffic safety and operation [16]. Results suggest the need for driver behavior analysis through the means of quantitative analysis. The current research is an attempt to fill this gap and contribute to the existing literature by (1) exploring the impact of fabricated content on speed choice behavior of drivers and (2) understanding observed and unobserved determinants of speed choice behavior in a compromised DMS condition. In particular, we test the following hypotheses:

- **Hypothesis 1:** Compromised DMS will destabilize the transportation network by changing the travel speed of drivers.
- **Hypothesis 2:** The response of drivers to a compromised DMS varies according to socioeconomic and character attributes of drivers.
- **Hypothesis 3:** The response of drivers to compromised DMS varies according to the character of bogus contents.

To corroborate the hypotheses, we develop a set of multivariate latent-based ordered probit models on 4,302 observations collected by Amazon Mechanical Turk between November and December 2018. For this study, we asked participants to state their speed choice preference considering a different set of forged DMS messages. The remainder of the article discusses the data collection, model specification, results of the analysis, main findings, and further research avenues.

## Data collection, description, and result

The core data used in this study were collected by the research team at Virginia Tech. We distributed a Stated Preference (SP) questionnaire through the Amazon Mechanical Turk among licensed drivers in 10 states including California, New York, Texas, Florida, New Jersey, Mississippi, Iowa, Virginia, Maryland, and North Carolina as well as the District of Columbia. Our main criterion to select the geographical distribution of the sample was the frequency of DMS hacking events in the US [16,17]. The data collection process happened between November and December 2018. We received 4,706 completed questionnaires, which are then reduced to 4,302 observations following the data cleaning process and elimination of erroneous and invalid responses.

The questionnaire is comprised of four sections:

- **Section 1:** This section captured the socioeconomic and demographic information of participants. We collected information on age, gender, household income, driving experience, and education level. Participant are also asked about their driving style in this section, which they could select (1) “anxious” (i.e., feelings of alertness and tension), (2) “Reckless and careless” (i.e., violations of safe driving norms), (3) “Angry and hostile” (i.e., tendency to act aggressively on the road), or (4) “Patient and careful” (i.e., planning ahead, paying attention, and being patient).
- **Section 2:** This section collected information on attitudes and preferences of participants toward (1) use of technology in their day-to-day commute, (2) traffic regulations, (3) adjacent traffic attentiveness, and (4) sense of direction.
- **Section 3:** This section measured whether and to what extent participants are familiar with DMS and how often they encounter and read DMS in their daily commute.
- **Section 4:** The last section investigates the response behavior of participants under different compromised DMS scenarios. Participants were given a set of questions to state their speed choice behavior under different realistic and fictitious scenarios. Scenarios are (1) S1: “Road Closure due to Police Activity,” (2) S2: “Heavy Traffic due to accident,” (3) S3: “Read the News Today, Oh Boy!” and (4) S4: “Zombies ahead run!” For instance, for S1: “Road Closure due to Police Activity” scenario, the statement included in the questionnaire reads as follows:

“Assume that you are driving on a highway to reach downtown with the speed limit of 60 mph. You come across a DMS that says "Road Closure Due to Police Activity." Please mark the following statements on a scale of extremely likely to extremely unlikely.”

We refer to S1 and S2 as realistic scenarios and S3 and S4 as fictitious scenarios. The fictitious content is designed to mimic the real world compromised DMS events [16], while the fabricated-realistic contents are conceivable to cause a higher negative impact.

While collecting the data, we noticed the sample is skewed toward younger, female, and low-income individuals, which is a common bias when using Amazon Mechanical Turk. To avoid this skew, we continuously compared the sample demographic information with population in each state using United States Census Bureau and Federal Highway Administration data and judged the significance of difference using the Student’s t-statistic. We controlled the demographic of our population by offering higher incentives for the underrepresented population and limiting the responses of the overrepresented population.

**Fig 1:**
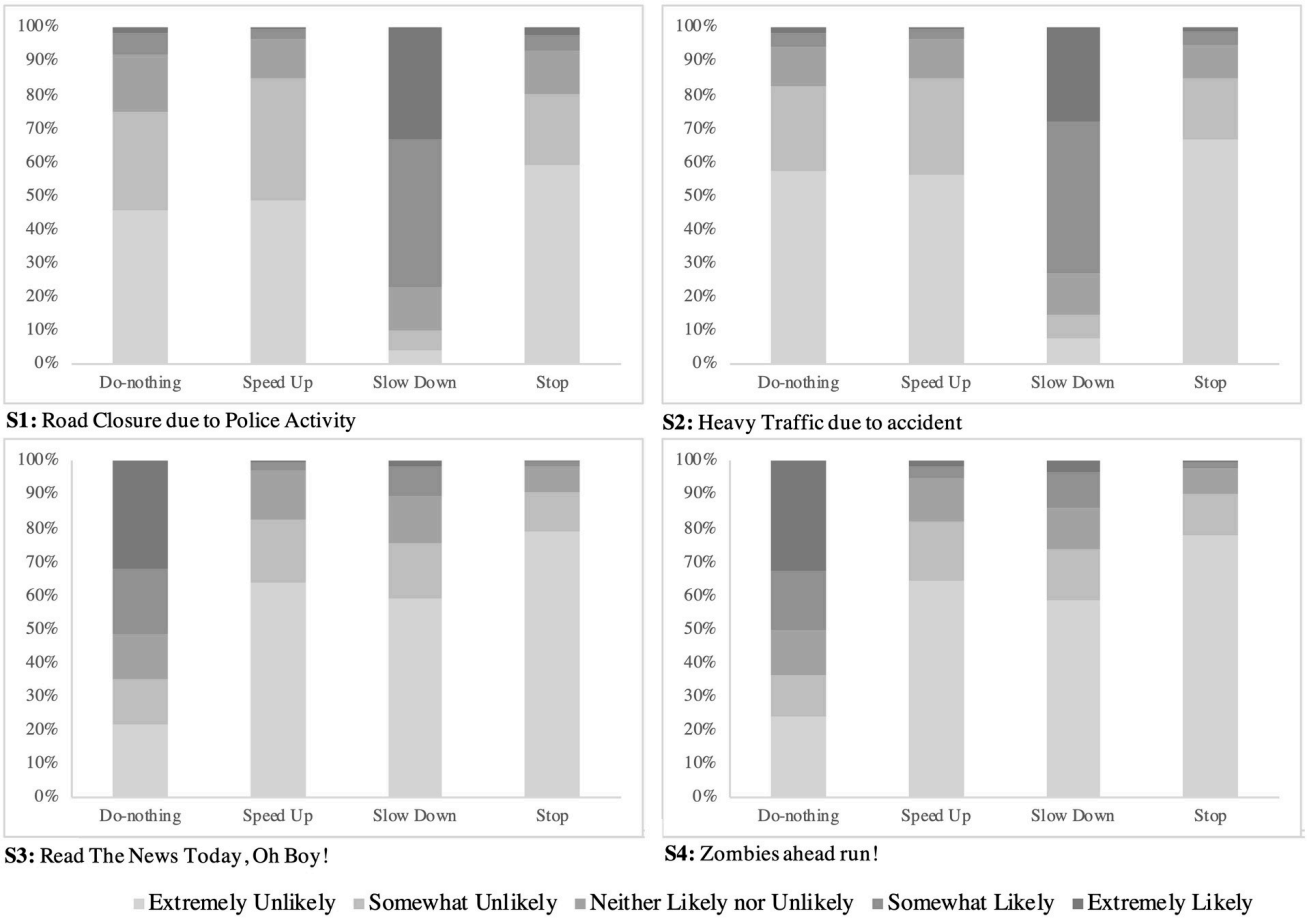
Road users speed choice behavior under a hacked DMS.

We encapsulate a preliminary description of data in the following:

- Out of 4,302 valid responses, 2,301 are female. Most of the participants are younger than 45 years, and 65% of them are white. Out of all participants, 2,257 have a degree of Bachelor’s or higher, and 30% of them hold a degree higher than Masters. The household income level of 2,131 participants is lower than $60,000, and 20% of them earn less than $30,000 per year.
- Of the participants, 2,635 participants have experienced an accident, and a quarter of those reporting an accident cited the reason as distracted driving. The frequency of “patient and careful” and “anxious” driving style is 70% and 23%, respectively.
- More than 70% of participants slow down under the “road closure due to police activity” scenario. Similar behavior is observed under the “heavy traffic due to accident” scenario. The behavior, however, is different in fictitious scenarios. About half of participants ignore “Read The News Today, Oh Boy!” and “Zombies ahead run!” contents. Fig 1 provides detailed information about the speed choice behavior of drivers in each scenario.

The description of the data used in the analysis is depicted in Table 1.

**Table 1:** Descriptive of the data used in the analysis.

Variable	Description	Mean	Std. Dev.
Female	1: Female; 0: Otherwise	0.53	0.50
Age	1: 18–24; 2: 25–34; 3: 35–44; 4: 45–54; 5: 55–64; 6: 65–84; 7: >85 years old	2.61	1.20
Young	1: If the participant is between 25 and 34 years old; 0:Otherwise	0.40	0.49
Driving experience	1: less than 1; 2: 1–5; 3: 610; 4: 11–15; 5: 16–20; 6: > 20 years	0.07	0.25
Little Driving experience	1: If driving experience is between1 and 5 years; 0: Otherwise	0.14	0.35
High Driving experience	1: If driving experience is more than 20 years; 0: Otherwise	0.32	0.47
Low Educated	1: If participant has an associate degree or lower; 0: Otherwise	0.48	0.50
Highly Educated	1: If participant has a degree above Bachelor’s; 0: Otherwise	0.52	0.50
Black	1: Participant is African-American; 0: Otherwise	0.09	0.29
White	1: Participant is White; 0: Otherwise	0.66	0.47
Single	1: Single, never married; 0: Otherwise	0.43	0.49
Impaired	1: Participant has a disability; 0: Otherwise	0.06	0.23
High Income	1: Income: above $90k; 0: Otherwise	0.24	0.42
Motorcycle/scooter	1: Yes; 0: Otherwise	0.01	0.08
Minivan/Van/MPV	1: Yes; 0: Otherwise	0.05	0.22
Single unit truck	1: Yes; 0: Otherwise	0.00	0.05
Rural	1: Rural area; 0: Otherwise	0.13	0.34
Urban	1: Urban area; 0: Otherwise	0.34	0.47
High Driving Hours	1: Driving between16 and20] hours per week; 0: Otherwise	0.07	0.26
Patient	1: Patient Driver; 0: Otherwise	0.70	0.46
Anxious	1: Anxious Driver; 0: Otherwise	0.23	0.42
Reckless	1: Reckless and careless Driver; 0: Otherwise	0.02	0.15
Angry	1: Angry and hostile Driver; 0: Otherwise	0.04	0.20
Involved in accident	1: Yes; 0: Otherwise	0.61	0.49
DMS_Familiar	Participant is 1: Not familiar; 2: Slightly familiar; 3: Moderately familiar; 5: Extremely familiar with DMS	3.96	1.02
DMS_Read	Participant 1: Never; 2: Sometimes; 3: About half the time; 4: Most of the time; 5: Always reads DMS in daily commute	4.28	0.89
DMS_Attention	Participant pays 1: Not at all; 2: Slightly; 3: Moderately; 4: Very; 5: Completely attention to DMS traffic information	4.31	0.82
DMS_Trust	Participant Trust in the DMS is from 0: not at all to 5: completely	4.09	0.82
High DMS_Trust	1:Participant Trust in the DMS is above 80% trust in DMS; 0: Otherwise	0.58	0.49
Rlying Tech	Participant relies on technology for daily trips from 1: Extremely Unlikely to 5: Extremely Likely	3.23	1.45
Trouble Direction	Participant has trouble understanding directions from 1: Extremely Unlikely to 5: Extremely Likely	2.18	1.17
Accomplished	Participant accomplishes more because of technology from 1: Extremely Unlikely to 5: Extremely Likely	4.03	0.92
Getting Bored	Participant is bored of driving from 1: Extremely Unlikely to 5: Extremely Likely	2.63	1.16
Up-To-Date	Participant is up-to-date with news from 1: Extremely Unlikely to 5: Extremely Likely	3.78	1.02
Using Blinker	Participant uses blinker when changing the lanes from 1: Extremely Unlikely to 5: Extremely Likely	4.64	0.73
Paying Attention	Participant pays attention to surrounding vehicles from 1: Extremely Unlikely to 5: Extremely Likely	4.70	0.62
Familiar Route	Participant prefers familiar route for day-to-day commute from 1: Extremely Unlikely to 5: Extremely Likely	4.30	0.86
Getting Lost	Participant are easily getting lost in an unfamiliar route from 1: Extremely Unlikely to 5: Extremely Likely	3.10	1.29
Complying	Participant complies with traffic regulations from 1: Extremely Unlikely to 5: Extremely Likely	4.52	0.74
Following	Participant drives the same way as others from 1: Extremely Unlikely to 5: Extremely Likely	3.36	1.09
Having Good Record	Participant has a good record of driving from 1: Extremely Unlikely to 5: Extremely Likely	4.46	0.81
Checking Traffic	Participant checks traffic before hitting the road from 1: Extremely Unlikely to 5: Extremely Likely	3.19	1.38
Trusting Tech	Participant trusts technology to assist in her travel from 1: Extremely Unlikely to 5: Extremely Likely	4.17	0.94

## Method and model

The speed choice falls into four categories of (1) Do nothing, (2) Speed up, (3) Slow down, and (4) Stop, which are answered in an ordinal scale with 1 to 5 Likert scale responses ranging from “1: extremely unlikely” to “5: extremely likely.” We, hence, develop four Multivariate Latent-Based Ordered Probit models to understand speed choice behavior in each scenario. This model is an extension of the Multivariate Probit Regression when the outcome variables have more than two categories and help (1) capture the correlation between the error terms of each speed choice and (2) embed unobserved latent variables.

As shown in Table 2, we examine the latent variables using explanatory factor analysis. This analytical method suggests three attitudinal latent factors describing the attitude of driver toward (1) Technology (Tech), (2) Driving Habit (Habit), and (3) Sense of Direction (Direction).

**Table 2:** Factor loading: Explanatory factor analysis.

Indicators	Attitudinal Latent Factors		
	Driving Habit	Tech	Direction Attitude
Use blinker when changing the lanes	**0.712**	0.187	0.012
Pay attention to vehicles around me	**0.794**	0.176	-0.068
Comply with traffic regulations	**0.706**	0.133	0.006
Good record of driving	**0.599**	0.114	-0.109
Accomplished because of technology	0.274	**0.527**	0.149
Trust technology to assist in travel	0.242	**0.649**	0.250
Rely on technology for daily trips	0.006	**0.627**	0.203
Check traffic before hitting the road	0.083	**0.350**	-0.036
Trouble understanding directions	-0.174	0.135	**0.595**
Get lost easily in an unfamiliar route	0.010	0.282	**0.844**
Prefer familiar routes	0.338	0.193	**0.329**
Driving is boring	-0.112	0.059	0.223
Up-to-date with News	0.266	0.116	-0.181
Driving the same way as the others	0.130	0.204	0.074
Trust drivers around	-0.059	0.075	-0.060

**Fig 2:**
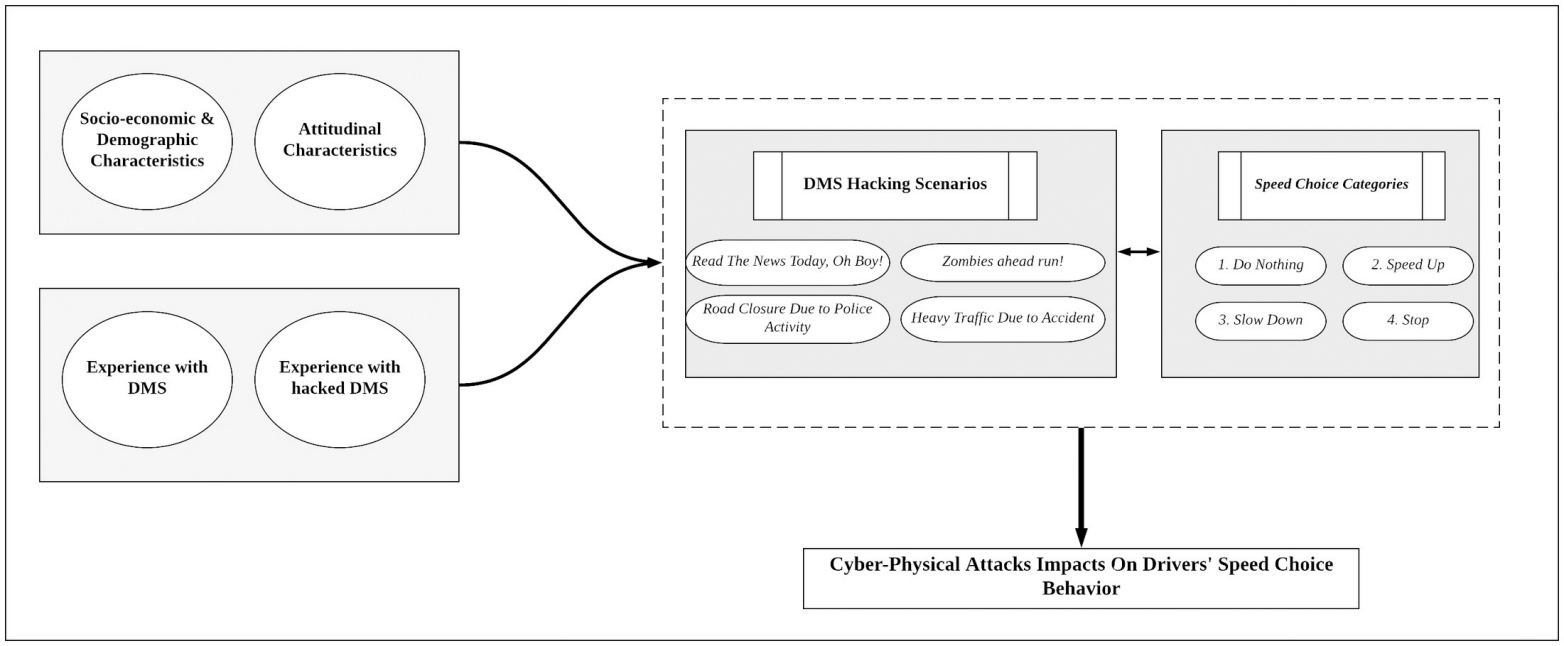
Conceptual modeling framework.

We used a multivariate ordered response structure in order to layout drivers’ speed change behavior under a compromised DMS. This approach has been previously used in the transportation literature [18,19]. This methodology enables mapping unobserved latent variables reported in Table 2 with a set of observed ordinal dependent variables. The underlying structure depicted in Fig 2 shows the use of latent variables as a covariance matrix while taking into consideration the correlation pattern between model’s background and outcome variables. Consider:

- *m* as an indicator for individual drivers (*m* = 1,2,3, …, *M*);
- *j* as an indicator for drivers-related attitudinal variables (*j* = 1,2,3, …, *J*); and
- *L*_*j*_ to be the response values for the attitudinal variable *j* where *L*_*j*_ belongs to (1,2,3, …, *L*_*j*_).

The unobserved latent propensity $\left(Y_{mj}^{*}\right)$ for each of the speed choice outcome variable (*Y*_*mj*_) can be written as Eq 1:

$$Y_{mj}^{*}\;=\;{\overset{´}{\beta }}_{j}x_{mj}+\varepsilon_{mj},\;{and\;Y}_{mj}\;=\;L\;if\;\tau_{j}^{l-1}\;<Y_{mj}^{*}<\tau_{j}^{l}$$ (1)

Where:

*x*_*mj*_: Vector of exogenous variables

*β*_*j*_: Vector of coefficients

*ε*_*mj*_: Standard normal error terms

$\tau_{j}^{l}$: Upper bound cut-point for response level *l*

The reader is referred to Seraj et al [20] for more information on the methodology.

To build the models, we pursue the following steps:

- We test the correlation between the dependent and independent variables using a Bivariate Probit Regression model to examine the correlates of speed choice behavior.
- We assess the significance of independent variables in the model using the student’s t-statistic and embed significant variables at a 90% confidence interval.
- We test the collinearity between variables and remove highly correlated variables with a lower impact on the goodness-of-fit of the model. We measure the goodness-of-fit by McFadden’s Pseudo R-Square, which ranges between 0 and 1. The closer McFadden’s Pseudo R-Square is to one, the better the model fits the data.

Tables 3 and 4 report the results of the modeling. As shown, McFadden’s Pseudo R-Square equals 0.66, 0.64, 0.53, and 0.79 for S1, S2, S3, and S4, respectively.

**Table 3:** Speed choice under realistic scenarios.

	S1: Road Closure due to Police Activity								S2: Heavy Traffic due to accident							
Variables	Do-nothing		Speed Up		Slow Down		Stop		Do-nothing		Speed Up		Slow Down		Stop	
	Coef.	z	Coef.	z	Coef.	z	Coef.	z	Coef.	z	Coef.	z	Coef.	z	Coef.	z
*Female*	-0.14***	-2.98	–		0.25***	6.61	–		–		–		0.13***	3.43	-0.09**	-2.37
*Age*	–		–		–		–		-0.08***	-4.41	–		–		–	
*White*	0.13**	2.4	–		-0.10**	-2.51	–		–		–		-0.14***	-3.52	–	
*Black*	-0.18**	-1.99	–		–		0.19***	3.12	-0.36***	-4.35	–		–		0.22***	3.2
*Low Educated*	–		–		–				-0.09**	-1.97	–		–		–	
*Rural*	–		–		–				–		-0.21***	-2.63	0.22***	3.88	–	
*Impaired*	-0.28***	-2.85	–		–		–		–		–		–		–	
*DMS_Read*	-0.15***	-5.52	–		–		-0.13***	-6.31	-0.12***	-4.84	–		–		–	
*DMS_Familiar*	–		-0.08***	-3.86	–		–		–		–		–			
*Driving experience*	–		–		–		-0.09***	-7.49	–		–		–			
*Involved in accident*	–		–		–		–		–		-0.19***	-3.49	–		-0.20***	-4.85
*Minivan/Van/MPV*	–		–		–		–		–		–		–		0.154*	1.76
*DMS_Trust*	-0.28***	-8.51	-0.27***	-8.91	0.18***	7.49	–		–		–		–		–	
*High DMS_Trust*	–		–		–		–		-0.21***	-4.44	–		–		–	
*DMS_Attention*	-0.28***	-8.85	-0.18***	-6.27	–		–		–		–		–		–	
*High Driving Hours*	0.16*	1.92	–		–		–		–		–		–		–	
*Patient*	-0.11**	-2.27	–		–		–		–		–		–		–	
*Reckless*	–		–		–		–		–		0.48***	3.04	–		–	
*Up-To-Date*	-0.041*	-1.83	–		–		–		–		–		–		–	
*Getting Bored*	–		–		–		–		0.09***	4.37	–		–		–	
*Take picture*	-0.05***	-2.65	–		0.04**	2.57	–		–		–		0.06***	4.25	–	
*Check radio*	-0.09***	-5.18	–		0.13***	8.85	–		–		–		0.07***	5.29	–	
*Look around*	–		–		0.16***	10.43	–		–		–		0.13***	9.27	–	
*Call/Text*	–		–		–		0.18***	11.31	–		–				0.28***	16.45
*Browsing*	–		–		–		0.11***	7.86	–		–		–		–	
*Route Change*	-0.34***	-16.64	–		0.22***	12.73	0.1***	6.13	-0.23***	-13.02	–		0.08***	5.15	–	
*Direction Attitude*	–		–		–		–		–		–		0.03*	1.94	–	
*Tech*	–		–		0.11**	2.33	–		–		–		–		–	
**Thresholds (cut-points)**																
*Cut 1*	-5.08***	-25.98	-2.22***	-14.25	0.61***	4.62	0.41***	3.56	-1.2***	-8.57	0.22***	4.65	-0.67***	-8.552	0.94***	17.62
*Cut 2*	-3.83***	-20.13	-0.73***	-4.85	1.13***	8.58	1.11***	9.71	-0.07	-0.529	1.71***	21.4	-0.25***	-3.276	1.68***	29.26
*Cut 3*	-2.67***	-14.29	0.61***	3.91	1.77***	13.28	1.85***	15.81	0.82***	5.929	3.07***	24.09	0.28***	3.576	2.45***	37.09
*Cut 4*	-1.58***	-8.29	1.57***	8.05	3.32***	23.25	2.48***	19.98	1.65***	11.087	4.09***	22.13	1.79***	21.41	3.2***	35.65
**Latent factors indicators**																
**Tech**			**Direction Attitude**													
Indicator	Coef.	z	Indicator	Coef.	z											
*Accomplished*	Constant		*Getting Lost*	Constant												
*Trust Tech*	1.33***	24.17	*Familiar Route*	0.19***	17.09											
*Relying Tech*	1.49***	20.56	*Trouble Direction*	0.46***	26.04											
*Checking Traffic*	0.71***	11.96	–													

Note:

(1) ***, **, * means significance at 1 percent, 5 percent, 10 percent level.

(2) McFadden’s Pseudo R-Square equals 0.66, 0.64 for scenario 1, and 2, respectively.

**Table 4:** Speed choice under fictitious scenarios.

	S3: Read The News Today, Oh Boy!								S4: Zombies ahead run!							
Variables	Do-nothing		Speed Up		Slow Down		Stop		Do-nothing		Speed Up		Slow Down		Stop	
	Coef.	z	Coef.	z	Coef.	z	Coef.	z	Coef.	z	Coef.	z	Coef.	z	Coef.	z
*Female*	–		–		–		–		–		-0.08*	-1.64	–		–	
*Age*	–		–		0.06***	2.89	–		–		–		–		–	
*Young*	–		–		–		–		-0.09**	-2.13	–		–		–	
*White*	0.12***	2.82	–		–		–		–		-0.19***	-3.52	–		-0.27***	-3.62
*Asian*	–		–		–		–		–		–		–		–	
*High Income*	–		–		–		–		–		-0.13**	-2.39	–		–	
*Highly Educated*	–		–		–		–		–		–		0.12***	2.91	–	
*Single*	–		–		0.12**	2.57	–		–		–		–		–	
*Urban*	–		–		–		0.19***	2.77	–		–		–		0.18***	2.59
*Driving experience*	0.06***	4.1	-0.08***	-5.516	–		–		–		–		–		–	
*Little Driving experience*	–		–		–				–		–		0.11*	1.89	0.22**	2.4
*High Driving experience*	–		–		–				0.13***	2.58	–		–		–	
*Motorcycle/scooter*	–		–		–		0.77**	2.25	–		–		–		–	
*Involved in accident*	–		–		–		-0.23***	-3.28	–		–		–		–	
*DMS_Trust*	–		–		–		-0.3***	-7.18	–		–		–		–	
*Anxious*	–		–		0.13***	2.71	–		–		–		–		–	
*Reckless*	–		–		–		0.46**	2.32	–		–		–		–	
*Single Unit Truck*	–		–		0.61*	1.8	–		–		–		–		–	
*Getting Bored*	–		–		0.05***	2.7	–		–		–		–		–	
*DMS_Familiar*	0.12***	5.88	–		–		–		–		–		–		-0.13***	-4.11
*DMS_Read*	–		-0.19***	-8.11	-0.1***	-4.24	–		0.1***	4.44	–		–		–	
*Take picture*	–		–		0.25***	13.91	0.19***	5.95	-0.16***	-11.01	–		0.14***	8.44	–	
*Call/Text*	–		–		0.19***	8.62	0.33***	8.42	-0.24***	-15.22	–		0.15***	8.89	0.22***	8.95
*Check radio*	–		–		0.27***	16.24	0.32***	8.75	–		–		–		–	
*Browsing*	–		–		–		0.1***	3.09	–		–		–		–	
*Driving Habit*	–		–		–		–		0.79***	14.68	–		–		–	
**Thresholds (cut-points)**																
*Cut 1*	-0.12***	-1.24	-0.69***	-6.122	1.76***	11.85	2.33***	9.29	-1.52***	-13.51	0.45***	7.83	1.25***	19.74	1.62***	8.88
*Cut 2*	0.39***	4.09	0.04***	0.358	2.48***	16.07	3.41***	11.72	-1.08***	-9.66	1.3***	19.57	1.89***	27.73	2.66***	12.56
*Cut 3*	0.79***	8.29	1.21***	9.951	3.23***	20.30	4.96***	13.79	-0.68***	-6.12	2.27***	28.27	2.47***	33.79	3.86***	15.27
*Cut 4*	1.35***	14.0	2.01***	13.991	4.22***	25.02	6.18***	14.36	-0.15	-1.30	2.77***	30.60	3.34***	39.81	4.71***	16.22
**Driving Habit Latent factor**																
*Use Blinker*	constant															
*Paying Attention*	0.94***	42.36														
*Complying*	1.01***	39.15														
*Having Good Record*	0.94***	33.59														

Note:

(1) ***, **, * means significance at 1 percent, 5 percent, 10 percent level.

(2) McFadden’s Pseudo R-Square equals 0.53, and 0.79 for scenario 3, and 4, respectively.

## Results and discussion

### Socioeconomic and attitude correlates

Looking at Scenario 1, it is found that drivers with higher trust in DMS, those who pay more attention to the traffic-related information, and those with a disability are likely to comply the fabricated-realistic information. However, white participants and those with long weekly commute hours are more likely to ignore the fabricated-realistic information. All significant explanatory variables have a negative correlation with increased speed, indicating that participants who are familiar with DMS are unlikely to speed up. We found female drivers, drivers with high trust in DMS, and tech-friendly drivers are likely to slow down in Scenario 1. The results also show that African-American drivers, drivers with a lack of sense of direction, and drivers with careful driving style are likely to stop.

Similar to Scenario 1, all variables except “Getting Bored” has a negative correlation with the do-nothing choice in Scenario 2. The results indicate that older drivers, drivers with lower education, and drivers with more attention to DMS are likely to comply the fabricated-realistic information. However, if the respondent finds driving a tedious activity, the chance of ignoring the fabricated-realistic information is higher for the respondent. We found female, white, and high-income drivers are unlikely to speed up in Scenario 2, while reckless and careless drivers are likely to speed up. The results also demonstrate that female drivers, drivers in rural areas, and drivers with a lack of sense of directions are likely to slow down. If the respondent was an African-Americans, males, and safe drivers have a higher chance of stopping in Scenario 2 than other groups.

Looking at Scenario 3, it is found that the tendency to slow down or stop is statistically more significant in our sample than do nothing or speed up. The results demonstrate that white drivers, drivers with long weekly commute hours, and drivers with DMS familiarity are likely to ignore Scenario 3. Compared with other speed choices (i.e., ignore, slow down, stop), participants with more driving experience, and those who read the content of DMS with more frequency are less likely to speed up under Scenario 3. All, but the “DMS_Read” variable were found to have a positive correlation to slowing down under Scenario 3. Drivers of a single unit truck, anxious drivers, single, and older drivers are more likely to slow down in Scenario 3. We also found drivers who read DMS more often in their commute are less likely to slow down. The results indicate that reckless and careless drivers, those who live in urban areas, and those who ride a motorcycle for most of their commute are more likely to stop under Scenario 3. Previous involvement in an accident and high trust in the DMS are factors that inhibit subjects from stopping when the DMS disseminate fictitious message.

The result indicates that participant reaction to the Scenario 4 is fairly similar to Scenario 4. Participants with more than 20 years of driving experience and those who read DMS content in their daily commute are more likely to ignore the fabricated information. However, young drivers between 25 and 34 years show a negative attitude toward the do-nothing choice. Under Scenario 4, highly educated drivers and those who have up to five years of driving experience have a higher tendency to slow down under Scenario 4. Three variables which have a negative association with the speeding up in Scenario 4 are “White,”“High Income,” and “Female.” Living in urban areas and having relatively low driving experience are also factors that contribute to the higher chance of stopping under Scenario 4. The slow down and stopping behavior could rise simply from subjects’ interests in abnormal and infrequent messages. Nonetheless, the sign would cause unexpected slowdown and stopping behavior with the potential to create unsafe traffic patterns.

**Fig 3:**
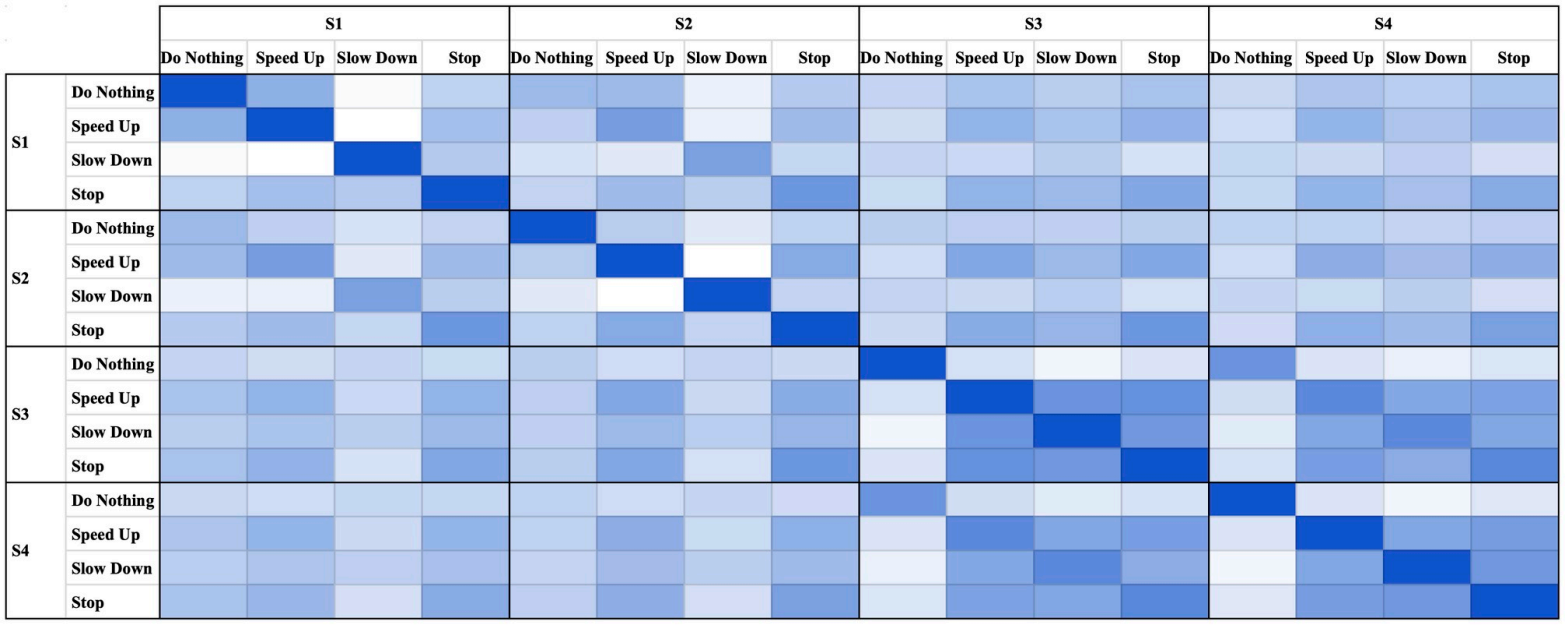
Heatmap of speed choice correlation matrix.

Here, we explore the correlation between speed behavior in and within four scenarios. Fig 3 depicts the correlation matrix. As shown, there is a strong correlation between similar choices of speed when comparing S1 and S2 with S3 and S4. For example, as indicated in Fig 3, drivers perceiving a slow down or stop are highly correlated under S1 and S2 or S3 and S4, showing similar speed variation behavior within scenarios under realistic or fictitious content.

The correlation estimates are not strong when assessing the results across the realistic and fictitious scenarios. Results show drivers conflicting attitudes toward the realistic scenarios compared to fictitious scenarios. In addition, among the four choices of speed, the do-nothing behavior indicates negative correlation across realistic and fictitious scenarios. In spite of these differences, drivers are corroborating similar patterns across all scenarios. This means speeding up, slowing down, and stopping is positively correlated across all scenarios. It is noteworthy to mention that the slowdown choice has the lowest positive correlation compared to speed up and stopping.

### Speed choice indicators

**Fig 4:**
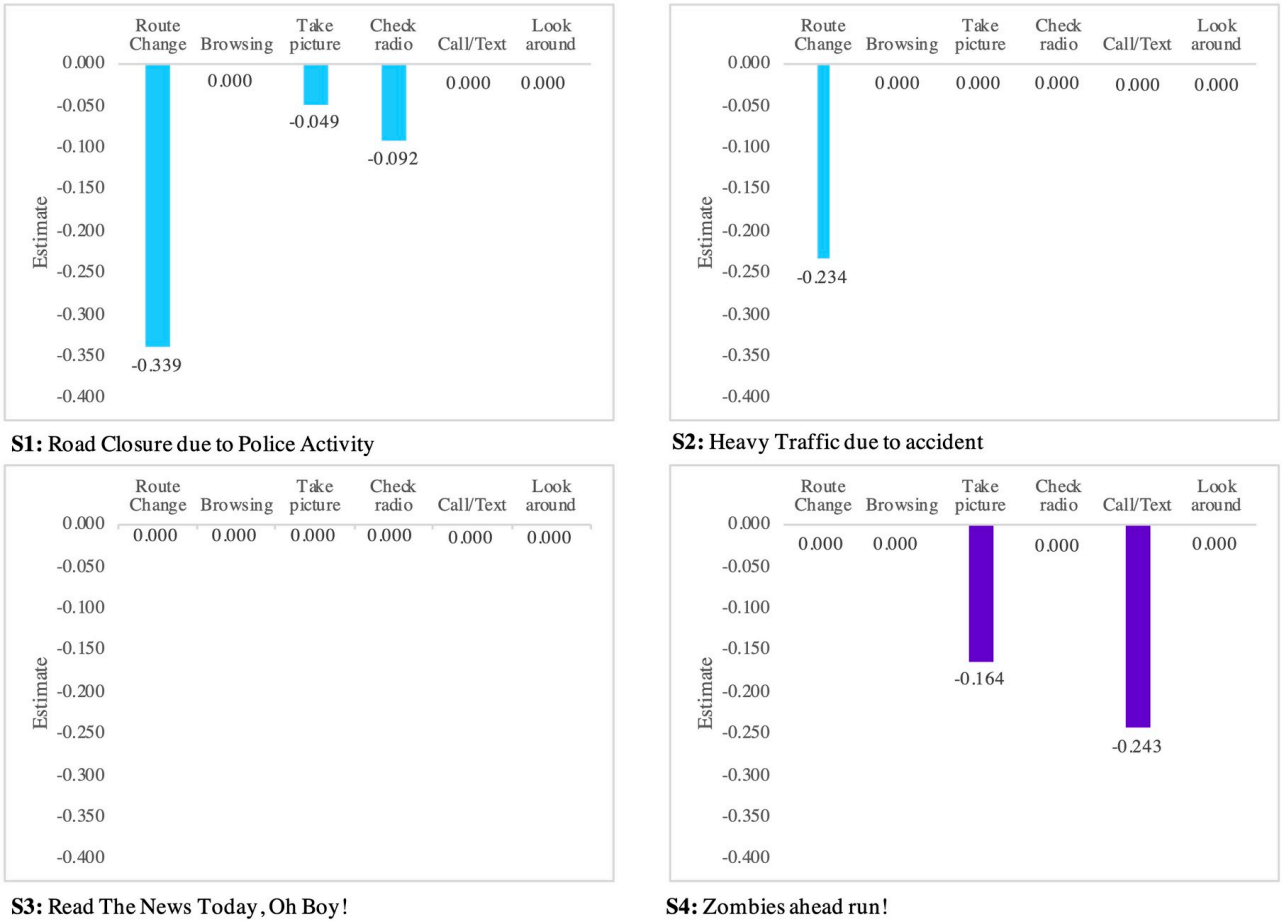
Indicators of do-nothing.

**Fig 5:**
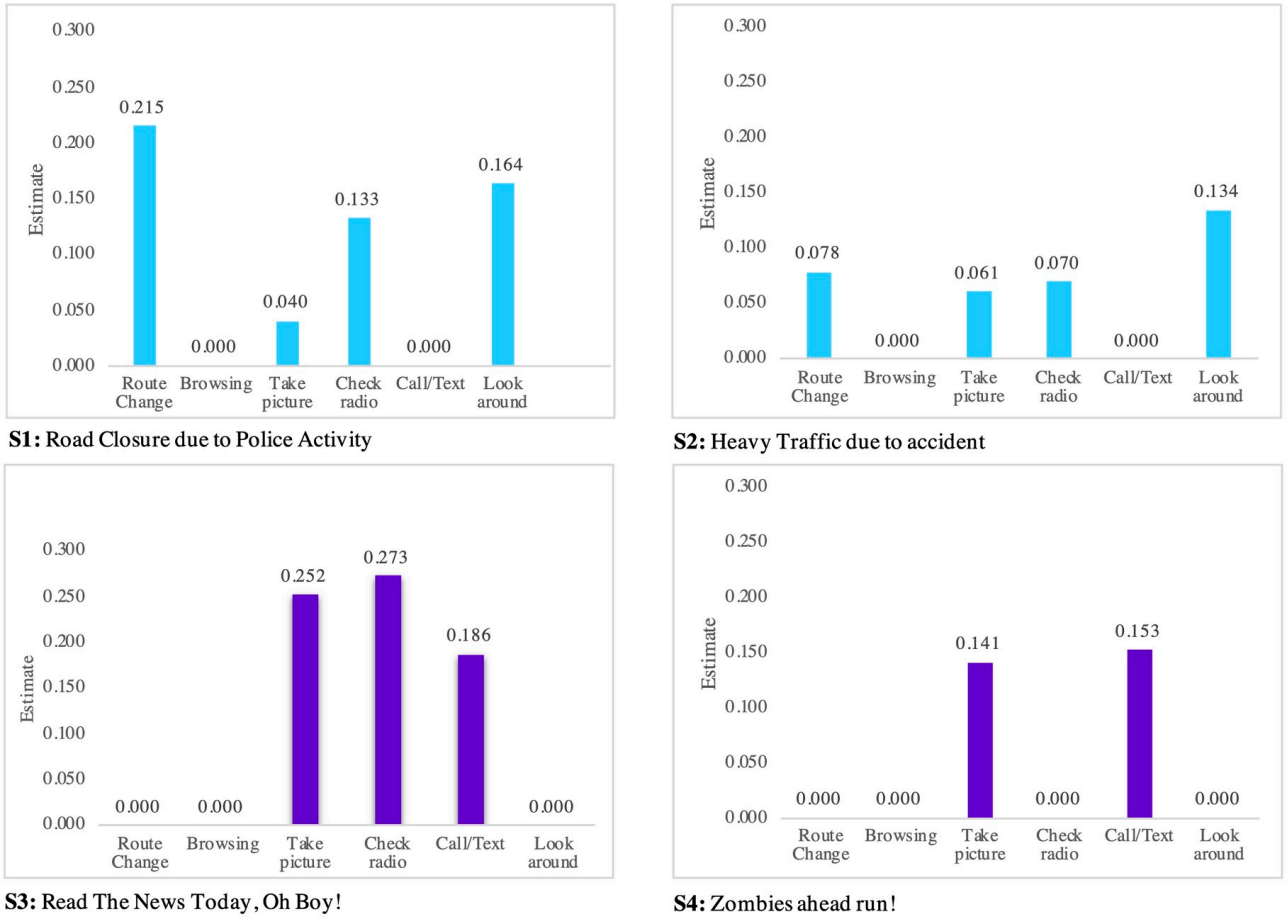
Causation factor of slow down behavior.

**Fig 6:**
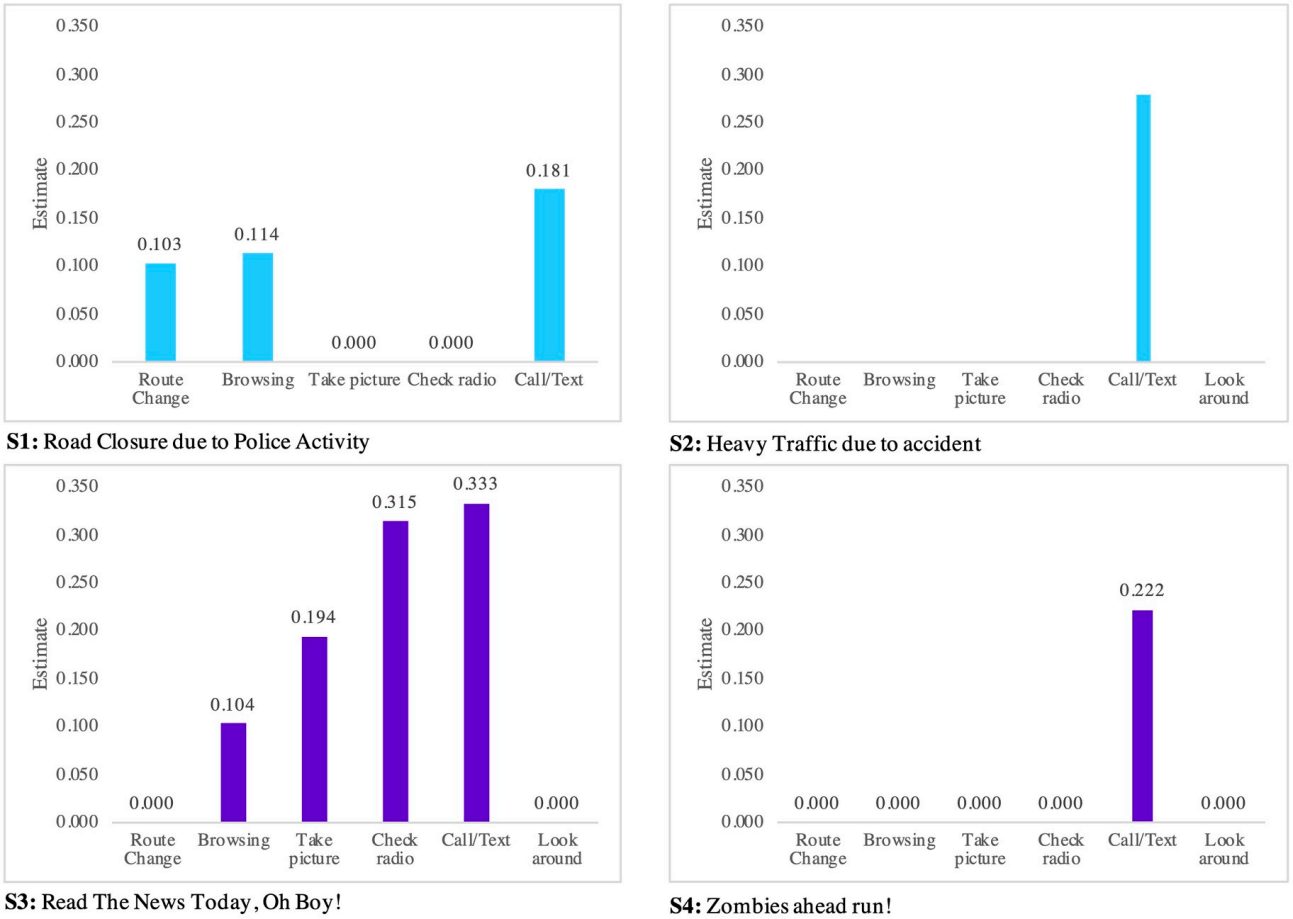
Causation factors of stopping behavior.

Here, we explore indicators impacting the speed choice behavior. The factors include (1) taking a picture, (2) checking the radio, (3) calling or texting, (4) browsing, (5) looking around, and (6) changing route. As represented in Tables 3 and 4, these indicators do not have a significant impact on speed up and only affect the do nothing, slow down, and stop speed choice behavior. The impact is depicted in Figs 4–6 for all scenarios.

Considering the do-nothing behavior, it is interpreted that drivers are likely to change their route when they read “Road Closure due to Police Activity” or “Heavy Traffic due to Accident” messages. As shown in Fig 4, drivers are potentially distracted by taking pictures, checking the radio, or calling or texting when they read “Road Closure due to Police Activity” or “Zombies Ahead Run” messages.

Considering the slowdown behavior, it is found that “Road Closure due to Police Activity” and “Heavy Traffic due to Accident” messages slow down drivers mostly because of (1) taking a picture, (2) checking radio, (3) looking around, and (4) changing the route. The slow down behavior, however, is typically generated by (1) taking a picture, (2) checking the radio, and (3) calling or texting under “Read the News Today, Oh Boy” and “Zombies Ahead Run” scenarios.

Considering the slowdown behavior, it is shown that calling or texting is one of the main reasons, if not the only one, to stop driving. However, as alluded to previously, drivers are likely to slow down rather than stop under fictitious content. This finding has a practical implication as it raises concerns about safety hazards.

## Summary and conclusion

The existing Intelligence Transportation System (ITS) literature has explored the speed choice behavior under advisory information [8–10]. Whilst previous research has been successful in examining the speed choice behavior of drivers, they overlooked the speed change behavior under compromised DMS. This study attempted to augment the ITS literature by exploring the speed choice behavior of drivers at the presence of fabricated advisory information. Not only did we examine the socioeconomic and attitude correlated with speed choice behavior in different scenarios, but we also delved into the indicators of speed choice behavior. This scrutiny provides valuable insight to policymakers to put in place effective policies to mitigate the risks that are associated with a compromised DMS. We summarize the key findings in the following:

- We found that females, African-Americans, drivers with a disability, elderly, and drivers with high trust in DMS are likely to comply with the fabricated messages. In contrary, Whites, those who drive longer hours (i.e., 16 to 20 hours per week), and those who see driving a tedious task are more likely to ignore the messages. Drivers who comply with traffic regulations and have a good driving record are likely to slow down under the messages. Female drivers and those who live in rural areas are more likely to slow down under fabricated advisory information.
- We investigated whether route divergence behavior and involvement in distractive activities could cause speed variation. The tryout divulged that (a) under realistic scenarios, visual distractions and route change behavior are activities that have the highest impact on slow down behavior, (b) under fictitious scenarios, calling or texting someone, taking a picture of the sign, and tuning the radio are distracting activities which cause drivers to slow down, and (c) under a fabricated message, the calling and texting activities have the highest causation impact on the stopping behavior. Sudden speed change behaviors could impose safety risks [21]. For instance, an unexpected slowdown or lane changing behaviors could increase the risk of a rear-end crash. The risk would be greater in high-volume traffic and highly congested roads.

We are aware of the limitation of the data due to the method of collection. The stated preference data might not seamlessly speak for drivers’ attitudes toward fabricated DMSs, and simulation-based data might be a better representative of this behavior. However, we argue that the results of this study are still of significant value to the research community due to the following reasons:

- Coming across a hacked DMS is not something that many people have experienced before. According to the results of this study, only 10% of the sample population interacted with a comprised DMS while driving. Surveying those participants’ behavior add immensely to the outcome of the research. Data scarcity as a result of simulation-based study would not necessarily guarantee the collection of a quality sample.
- While a simulation-based study can reveal the behavior of the driver, the sample population is less diverse compared to the web-based study. Driving simulation is a robust setting to capture drivers’ speed change behavior under a controlled environment; however access to the participants are limited to the geographic area of the study location. In other words, simulation-based data would not be as diverse as the web-based data.
- A simulation based study is generally more expensive than a web-based study causing limited interest from research teams to increase the sample population size. For the purpose of this original study, it was vital for the research team to analyze large, diverse, and geographically spread population sample to draw conclusions based off larger evidence.
- We deemed web-based approach as the best method of collecting data for this original study since there were no previous studies to provide prior knowledge. This study would fill the gap and set a baseline for future research studies to better understand the drivers speed change behavior at the compromised DMS. This prior knowledge would help research teams to assemble a sample population of participant’s characteristics with the intent to produce better results.
